# *Histoplasma* Seropositivity in TB Patients in The Gambia: Evidence to Drive Research on a High-Priority Fungal Pathogen

**DOI:** 10.1093/ofid/ofad510

**Published:** 2023-10-13

**Authors:** Tessa R Cornell, Dawda Jobe, Simon Donkor, Daniel G Wootton, Gina Pinchbeck, Jayne S Sutherland, Claire Elizabeth Scantlebury

**Affiliations:** Institute of Infection, Veterinary and Ecological Sciences (IVES), University of Liverpool, Liverpool, UK; Vaccines and Immunity Theme, MRC Unit The Gambia, London School of Hygiene and Tropical Medicine, Fajara, The Gambia; Vaccines and Immunity Theme, MRC Unit The Gambia, London School of Hygiene and Tropical Medicine, Fajara, The Gambia; Institute of Infection, Veterinary and Ecological Sciences (IVES), University of Liverpool, Liverpool, UK; NIHR Health Protection Research Unit in Emerging and Zoonotic Diseases, University of Liverpool, Liverpool, UK; Institute of Infection, Veterinary and Ecological Sciences (IVES), University of Liverpool, Liverpool, UK; Vaccines and Immunity Theme, MRC Unit The Gambia, London School of Hygiene and Tropical Medicine, Fajara, The Gambia; Institute of Infection, Veterinary and Ecological Sciences (IVES), University of Liverpool, Liverpool, UK

**Keywords:** *Histoplasma*, seroprevalence, The Gambia, tuberculosis

## Abstract

**Background:**

Inclusion of *Histoplasma* in the World Health Organization's first Fungal Priority Pathogens List under “high-priority” fungal species highlights the need for robust surveillance of *Histoplasma* spp. in endemic and underrepresented regions. Despite increasing reports of histoplasmosis in Africa, data on the burden of this fungal disease are sparse in The Gambia. This baseline study examined the human seroprevalence of anti-*Histoplasma* antibody in a TB patient group in The Gambia, explored associations between seropositivity and demographic and clinical variables, and proposes future research directions.

**Methods:**

Biobanked plasma samples were selected from active TB cases with variable HIV infection status. Latex agglutination tests were performed on samples from 52 study participants to detect the presence of anti-*Histoplasma* antibodies. Potential risk factors for *Histoplasma* exposure were explored using logistic regression analysis.

**Results:**

The sample seroprevalence of anti-*Histoplasma* antibody was 28.8% (n = 15/52; 95% CI, 17.1%–43.1%). Multivariable logistic regression analysis identified a statistically significant association between *Histoplasma* seropositivity and age (odds ratio, 0.91; 95% CI, 0.84–0.98; *P* = .008).

**Conclusions:**

This baseline study provides evidence of *Histoplasma* seropositivity in TB patients in The Gambia and explores risk factors for exposure. The small sample size and use of the LAT in TB and HIV-positive patient groups are significant study limitations. Future research directions are proposed to ascertain the burden of *Histoplasma* in general and patient populations and explore the context-specific risk factors for exposure and infection in The Gambia.

*Histoplasma* spp. are thermally dimorphic fungi with widespread global distribution. Human histoplasmosis has been associated with the phenotypic varieties *Histoplasma capsulatum* var. *capsulatum* and *H. capsulatum* var. *duboisii*. Recent inclusion in a high-priority group of fungal pathogens by the World Health Organization (WHO) highlights the need for more robust surveillance of *Histoplasma* spp. in endemic regions [1]. In The Gambia, research efforts are warranted to examine the current distribution and annual incidence rates of *Histoplasma* spp., the susceptibility of immunocompromised patient populations to histoplasmosis, and context-specific risk factors. These efforts will inform disease prevention and control strategies, antifungal treatment approaches, and public health interventions.

In Central and South America, disseminated histoplasmosis is recognized as a major defining disease presentation of patients with advanced HIV disease (AHD) [2, 3]. In this setting, incidence and mortality rates of opportunistic infections in patients with HIV infection have demonstrated a comparable or higher frequency of histoplasmosis compared with that of tuberculosis (TB) [4–6]. Furthermore, the clinical presentation and diffuse miliary radiographic pattern of disseminated histoplasmosis can resemble pulmonary TB, leading to misdiagnosis and lower clinical index of suspicion of histoplasmosis [7–10], in addition to underreporting of histoplasmosis and TB coinfections [11, 12].

*H. capsulatum* var. *duboisii* variety is described by the majority of histoplasmosis case reports in West Africa, with individuals presenting with cutaneous or bone lesions [13]. Case reports across West Africa [13] provide baseline data to support a more robust investigation of the epidemiological and clinical characteristics of *Histoplasma* spp. in this geographic region. Among 2 patient groups with HIV infection in Nigeria and Ghana, *Histoplasma* infection prevalence rates of 7.7% (n = 76/988) and 4.7% (n = 5/107), respectively, were measured by antigen test [14, 15]. In addition, in Nigeria, a histoplasmosis prevalence of 12.7% (n = 27/213) was reported in a patient population with presumptive pulmonary TB by antigen test or polymerase chain reaction (PCR) [16]. Prevalence was significantly lower in confirmed TB patients (n = 7/94, 7.4%) compared with unconfirmed TB patients (n = 20/119, 16.8%). These findings highlight that histoplasmosis surveillance is also pertinent in immunocompetent groups and in patients with clinical symptoms suggestive of TB with or without a confirmed TB diagnosis. Furthermore, the majority of histoplasmosis case reports in West Africa are described in patients with HIV-negative status [13, 17, 18].

Compared with TB, for which established research programs exist, no studies to date have examined the burden of *Histoplasma* in The Gambia. Evidence of histoplasmosis in The Gambia is limited to 2 historic case reports [19–21], thus highlighting the need to prioritize research on *Histoplasma* spp. in this context.

The current study utilized a subset of plasma samples and demographic and clinical data previously collected during a longitudinal cohort study in the Greater Banjul area, The Gambia. The primary objectives of this study were (i) to estimate the human seroprevalence of anti-*Histoplasma* antibody in a TB patient group in The Gambia using a latex agglutination test (LAT) and (ii) to explore associations between *Histoplasma* seropositivity on LAT and demographic and clinical variables in this patient group.

## METHODS

### Parent Study and Ethics Approval

A longitudinal cohort study with nested case–control studies was conducted from 2006 to 2012 as part of the multisite Gates Grand Challenge Study (GC6–74) [22]. Study participants included active TB cases (n = 447) recruited from the Greater Banjul area at the MRC Gambia TB clinic. Patients aged ≥15 years who were newly diagnosed with sputum smear–positive TB and living in the Greater Banjul area for >3 months were eligible to enroll as TB index cases. A full clinical history, physical examination, sputum culture, and chest radiograph were performed. Venous blood samples were collected and processed to investigate correlates of protective immunity and biomarkers of TB disease, and aliquots of plasma were stored at −20°C.

### Patient Consent

Written informed consent to all study interventions in the parent study and sample storage for future processing was obtained from all study participants. Ethical approval for sample collection and storage was granted by the Gambia Government/MRC Joint Ethics Committee (SCC 1034).

### Sample Selection and Serological Testing

From TB index cases with available plasma samples for secondary analysis (n = 440/447, 98.4%), a subset was selected (n = 52/440, 11.8%) for this study. Approximately half of participants demonstrating HIV-positive status in the original sample were purposefully selected for inclusion in this study subset, of whom 42.3% (n = 22/52) demonstrated HIV-positive status. Plasma samples were thawed, heat-treated (in a water bath at 56°C for 30 minutes), and analyzed using an IMMY Latex Agglutination *Histoplasma* test (LAT; Norman, OK, USA), performed as per manufacturer guidelines for each thawed, heat-treated sample [23]. In accordance with IMMY guidelines, a graduated scale of reaction strengths was used to assign test results from negative (−) to 4+. Positive and negative controls had to demonstrate ≥2+ and <1+ reaction strengths, respectively. Samples assigned a ≥2+ reaction strength were considered to be presumptive evidence of active or recent *Histoplasma* exposure [24–26]. Reaction strengths were assigned by 2 independent observers. If there were discrepancies between assigned results, samples were subjected to 1 repeat LAT and interpreted concurrently by both observers.

### Seroprevalence Estimation

The apparent prevalence of *Histoplasma* seropositivity in the sample population was determined based on LAT results. True seroprevalence was estimated using published sensitivity and specificity values for a histoplasmin-sensitized LAT of 62% and 97%, respectively [27]. Epitools interface and Clopper-Pearson (exact) tests were employed to determine 95% CIs (https://epitools.ausvet.com.au/trueprevalence) [28].

### Statistical Analysis

Demographic and clinical variables attached to samples were selected for analysis based on case reports describing clinical signs of histoplasmosis and evidence on clinical risk factors for *Histoplasma* infection or exposure, or evidence on *Histoplasma* transmission dynamics.

Descriptive statistics were used to analyze demographic and clinical characteristics of the selected subset of participants. The Mann-Whitney *U* test was applied to compare age, sex, and HIV infection status frequency distributions between the original sample (n = 440) and the selected subset (n = 52/440, 11.8%).

The Pearson chi-square test of association (χ^2^) was applied to examine univariable associations between *Histoplasma* seropositivity and selected variables. Odds ratios (ORs) with 95% CIs and associated *P* values were calculated.

Variables with a *P* value <.20 on univariable analysis were selected for testing in a multivariable logistic regression model with serostatus as the binary outcome. A manual backwards-stepwise approach was applied to eliminate variables. Variables were included in the model based on a cutoff value of *P* < .05. Random effects were included to explore the effect of clustering at both Local Government Area (LGA) and district levels.

## RESULTS

### Study Population

The selected subset of participants (n = 52/440, 11.8%) had a median age (interquartile range [IQR], range) of 34 (26.0–44.8, 17–65) years and comprised 34 males (65.4%). Eight ethnic groups were represented, with the majority of participants identifying as Jola (n = 20/52, 38.5%) or Mandinka (n = 15/52, 28.8%). Participants resided in 2 LGAs and 4 districts within the Greater Banjul area. The median number of contacts per household (range) was 3.5 (0–26).

In the original sample, a positive HIV status was recorded in 9.8% (n = 43/440) of participants, and HIV status demonstrated a statistically significant association with age (1.04; 95% CI, 1.02–1.07; *P* < .001). Frequencies of participant-reported clinical symptoms are reported by Table 1. Interquartile ranges for measured hematological parameters are within normal reference ranges for adult populations in West Africa [29, 30].

**Table 1.** Univariable Logistic Regression Analysis Exploring Associations With *Histoplasma* Seropositivity Among TB Index Cases in the Greater Banjul Area, The Gambia

Variable	Frequency, No. (%), Total n = 52	Median (Interquartile Range)	*Histoplasma* Seropositive, No. (%), Total n = 15	*Histoplasma* Seronegative, No. (%), Total n = 37	Odds Ratio (95% CI)^a^	*P* Value^a^
Demographic						
Sex						
Male	34 (65.4)	-	8 (23.5)	26 (76.5)	1.0	…
Female	18 (34.6)	…	7 (38.9)	11 (61.1)	0.48 (0.14–1.66)	.25
Age, y	-	34.0 (26.0–44.8)	-	-	0.91 (0.84–0.98)	.008*
Ethnic group						
Mandinka	15 (28.8)	…	6 (40.0)	9 (60.0)	1.0	…
Wolof	4 (7.7)	…	0 (0.0)	4 (100.0)	0.00 (0.00)	1.00
Fula	8 (15.4)	…	1 (12.5)	7 (87.5)	0.21 (0.02–2.22)	.20
Jola	20 (38.5)	…	7 (35.0)	13 (65.0)	0.81 (0.20–3.22)	.76
Other	5 (9.6)	…	1 (20.0)	4 (80.0)	0.38 (0.03–4.23)	.43
Clinical						
HIV status						
Negative	30 (57.7)	…	12 (40.0)	18 (60.0)	1.0	…
Positive	22 (42.3)	…	3 (13.6)	19 (86.4)	0.24 (0.06–0.98)	.047*
Chest x-ray findings^b^						
Minimal disease	4 (7.7)	…	1 (25.0)	3 (75.0)	1.0	…
Moderatelyadvanced	26 (50.0)	…	7 (26.9)	19 (73.1)	1.11 (0.10–12.47)	.94
Far advanced	16 (30.8)	…	7 (43.8)	9 (56.3)	2.33 (0.20–27.57)	.50
No x-ray	6 (11.5)	…	0 (0.0)	6 (100.0)	0.00 (0.00)	.99
White blood cells, ×10^9^/L	…	6.1 (4.9–8.0)	…	…	1.14 (0.86–1.51)	.37
Granulocytes, %	…	63.9 (54.7–71.7)	…	…	1.01 (0.96–1.06)	.81
Lymphocytes, %	…	28.7 (22.1–36.8)	…	…	0.99 (0.94–1.05)	.74
Monocytes, %	…	6.3 (5.2–7.3)	…	…	1.03 (0.79–1.33)	.85
Platelets, ×10^9^/L	…	335.5 (252.8–393.3)	…	…	1.00 (1.00–1.01)	.46
Cough						
No (no cough or <2 wk)	0 (0.0)	…	…	…	…	…
Yes (>2 wk)	39 (75.0)	…	14 (35.9)	25 (64.1)	1.0	…
Unknown	13 (25.0)	…	1 (7.7)	12 (92.3)	0.15 (0.02–1.27)	.08**
Night sweats						
No	40 (76.9)	…	14 (35.0)	26 (65.0)	1.0	…
Yes	1 (1.9)	…	0 (0.0)	1 (100.0)	0.00 (0.00)	1.00
Don’t know	11 (21.2)	…	1 (9.1)	10 (90.9)	0.19 (0.02–1.60)	.13**
Fever/chills						
No	40 (76.9)	…	14 (35.0)	26 (65.0)	1.0	…
Yes	1 (1.9)	…	0 (0.0)	1 (100.0)	0.00 (0.00)	1.00
Don’t know	11 (21.2)	…	1 (9.1)	10 (90.9)	0.19 (0.02–1.60)	.13**
Weight loss						
No	34 (65.4)	…	13 (38.2)	21 (61.8)	1.0	…
Yes	7 (13.5)	…	1 (14.3)	6 (85.7)	0.27 (0.03–2.50)	.25
Don’t know	11 (21.2)	…	1 (9.1)	10 (90.9)	0.16 (0.02–1.41)	.099**
Hemoptysis						
No	9 (17.3)	…	2 (22.2)	7 (77.8)	1.0	…
Yes	31 (59.6)	…	12 (38.7)	19 (61.3)	2.2 (0.39–12.47)	.37
Don’t know	12 (23.1)	…	1 (8.3)	11 (91.7)	0.32 (0.02–4.20)	.38
Environmental						
Local Government Area						
Brikama	25 (48.1)	…	7 (28.0)	18 (72.0)	1.0	…
Kanifing	27 (51.9)	…	8 (29.6)	19 (70.4)	1.08 (0.33–3.60)	.90
District						
Kanifing	27 (51.9)	…	8 (29.6)	19 (70.4)	1.0	…
Kombo Central	4 (7.7)	…	2 (50.0)	2 (50.0)	2.38 (0.28–19.9)	.43
Kombo North	18 (34.6)	…	4 (22.2)	14 (77.8)	0.68 (0.17–2.71)	.58
Kombo South	3 (5.8)	…	1 (33.3)	2 (66.7)	1.19 (0.09–15.04)	.89
No. of household contacts	-	3.5 (1–8.5)	-	-	0.98 (0.88–1.10)	.77
Season						
Dry (November–May)	…	-	9 (26.5)	25 (73.5)	1.0	
Wet (June–October)	…	…	6 (33.3)	12 (66.7)	1.39 (0.40–4.81)	.60

Abbreviations: OR, odds ratio; TB, tuberculosis.

^a^ORs, 95% CIs, and *P* values were calculated using IBM SPSS Statistics 25 software.

^b^Chest x-ray finding categories based on descriptors by Falk et al. (1969) [37].

^*^ *P* < .05.

^**^ *P* < .20.

No statistically significant differences were measured between age or sex distributions of the original sample and the selected subset. HIV-positive status was significantly higher in the subset (*P* < .001) due to the sample selection criteria applied.

### Seroprevalence Results

An apparent seroprevalence of 28.8% (n = 15/52; 95% CI, 17.1%–43.1%) was measured. The estimated true seroprevalence in this subset, adjusting for published LAT sensitivity and specificity results, was calculated as 43.8% (95% CI, 25.0%–66.8%) [28], which reflects the low sensitivity value of the LAT; 55.8% (n = 29/52) of samples were subject to a repeat LAT to achieve interobserver agreement.

### Logistic Regression Analysis

An exploratory univariable logistic regression analysis identified statistically significant associations (*P* < .05) between LAT result and both age (OR, 0.91; 95% CI, 0.84–0.98; *P* = .008) and HIV-positive status (OR, 0.24; 95% CI, 0.06–0.98; *P* = .047) (Table 1).

The final model contained only age as a statistically significant main effect (OR, 0.91; 95% CI, 0.84–0.98; *P* = .008). The model demonstrated that the odds of seropositivity decreased by 9.5% for every 1-year increase in participant age.

No evidence of clustering by district or LGA was demonstrated (variance = 0.0 SE 0.0). Regression coefficients, z-ratios, and *P* values of the age variable were comparable for single- and multilevel models.

## DISCUSSION

### Main Outcomes and Current Evidence

This study describes the seroprevalence of anti-*Histoplasma* antibody in a TB patient group residing in the Greater Banjul area, The Gambia, and explores associations between seropositivity and potential demographic and clinical risk factors. This is the first exploratory investigation of the level of *Histoplasma* exposure in The Gambia to help inform future research directions.

With 28.8% seroprevalence of *Histoplasma* exposure demonstrated in this study, we provide baseline data to support further investigation of the burden of *Histoplasma* in The Gambia in both HIV and non-HIV patients presenting with symptoms suggestive of TB. This study outcome supports the WHO's message in the First Fungal Priority Pathogens List (FPPL) [1] that robust research is required (i) to ascertain histoplasmosis incidence rates and distribution in underrepresented regions and (ii) to explore risk factors for *Histoplasma* exposure and infection in order to define high-risk demographic and patient groups. Literature on human histoplasmosis in The Gambia is limited to 2 historic case reports, which describe a child presenting with a lesion of the femur and cutaneous lesions [19, 20] and adenopathy and oral lesions in an adult residing in the United Kingdom but with a recent travel history to The Gambia [21]. These case reports did not describe the individuals’ medical history, identification of coinfections, or immunocompetence at the time of diagnosis. In addition, environmental risk factors for *Histoplasma* exposure in The Gambia were not examined. Case reports of histoplasmosis in nearby Senegal (n = 3), Guinea-Bissau (n = 1), and Mali (n = 2), diagnosed by direct microscopy, culture, or PCR technique, describe immunocompetent and -compromised individuals presenting with cutaneous, subcutaneous, oral and bone lesions, adenopathy, and disseminated infections [9, 31–35].

In Nigeria, cross-sectional multisite studies have examined *Histoplasma* infection and skin sensitivity in patient groups with variable HIV or TB status [16, 36]. One study reported *Histoplasma* infection prevalence values of 12.7% (n = 27/213) across the study population and 7.4% (n = 7/94) among participants with confirmed TB (sputum-positive by GeneXpert) using antigen testing and/or PCR, respectively [16]. A second study demonstrated a positive skin test for *Histoplasma* antigen in 4.4% (n = 32/735) of participants [36]. Neither study demonstrated significant associations between *Histoplasma* infection or positive skin test and HIV status on univariable analyses. Further prospective research examining the burden of *Histoplasma* in defined sample populations is required across West Africa to complement current evidence in this geographic region [14].

Demographic, clinical, and environmental variables explored by logistic regression analysis encompassed established and theoretically plausible epidemiologic risk factors for *Histoplasma* exposure. A significant association was identified between *Histoplasma* seropositivity and age. Evidence exploring age as a risk factor for *Histoplasma* exposure or infection is not conclusive. In Nigeria, no significant association was identified between age and *Histoplasma* infection status on univariable analysis in a patient group with presumptive pulmonary TB and with an age range of 18 to 81 years (mean, 39 ± 14 years) [16]. The focus of observational studies to date has been primarily to explore demographic and clinical risk factors for *Histoplasma* infection in patient groups with defined immunocompromised status or coinfections. As such, sample populations reflect the demographic profile of groups susceptible to the disease(s) under examination, rather than of a general population. Exposure and infection rates in apparently healthy populations and environmental risk factors should also be explored to ascertain the odds of exposure in younger and currently underrepresented age categories. In Kenya, a cross-sectional study examining anti-*Histoplasma* seropositivity in a general population measured significantly higher seropositivity in participants aged 15–24 years (compared with 25–35 years) on multivariable analysis [38]. Further research is warranted to determine whether the current study outcome reflects increased exposure to potential risk factors or immunocompetence variability in younger age categories.

On univariable analysis, the odds of *Histoplasma* seropositivity were significantly higher in patients with HIV-negative status, but this was not significant on multivariable analysis. As age was associated with both HIV status (in original and subset samples) and *Histoplasma* seropositivity, the variable may be a confounding factor and may also have affected the interpretation of other variables. The increased odds of HIV-positive status and concurrently decreased odds of *Histoplasma* seropositivity with age might also indicate a reduced ability of HIV-positive individuals to mount an immune response to *Histoplasma*, with subsequent false-negative results on LAT. Results concur with current evidence from West Africa that the majority of histoplasmosis case reports are described in HIV-negative patients [13]. Sample selection was stratified by HIV infection status, with 42.3% of participants demonstrating HIV-positive status. This statistic is significantly higher than the expected HIV infection prevalence in a patient group with confirmed TB (∼10%–15%). Despite the selection approach, HIV status may not be sufficiently powered to explore this variable as a risk factor. Additional studies using HIV status as the enrollment factor would enable a focused analysis of opportunistic fungal infections in patients with HIV infection. Importantly, this study is examining exposure rather than infection, and assessment of participant immunocompetence is restricted to reported bloodwork parameters and clinical symptoms. Furthermore, an examination of the impact of HIV infection on anti-*Histoplasma* antibody production has revealed significantly lower antibody detection in disseminated histoplasmosis cases with, compared with without, AHD [39], which can lead to an underestimation of seropositivity in HIV-positive individuals.

### Study Limitations

Interpretation of results is restricted by the limited size and scope of the data set. Variables under examination may not be sufficiently powered to determine their association with the odds of *Histoplasma* exposure, and clinical symptom data were not recorded for all participants. Furthermore, *Histoplasma* was not the focus of the original study; thus variables captured by the index case recruitment form were not designed to capture clinical risk factors or symptoms associated with *Histoplasma* exposure or infection. Temporal data relating to the timing of *Histoplasma* exposure are not available due to the cross-sectional study design; thus, considering the limited size of the data set, strong conclusions should not be made based on measured associations between serostatus and clinical indicators at presentation. The environmental data collected were limited to participant address and number of household contacts, and all participants resided within the primarily urban Greater Banjul area (for >3 months), thus preventing exploration of the effect of inter-regional environmental variations on seropositivity.

Clinical symptoms captured by the recruitment form were nonspecific and respiratory symptoms associated with pulmonary TB. Thus, nonrespiratory clinical presentations of histoplasmosis, including cutaneous and bone lesions, are not captured.

Limitations of the LAT are documented, including low test sensitivity and the occurrence of false-negative results in individuals tested before the IgM antibody response to *Histoplasma* has mounted (<2 weeks postexposure) and after the response has diminished [24, 26, 40]. A significant limitation of the study is the utilization of the LAT in HIV-positive patients, which comprise 42.3% of study participants. As per WHO (2020) guidelines pertaining to the management of disseminated histoplasmosis in people with HIV, antigen detection assays are recommended as diagnostic tools based on current evidence of their high diagnostic accuracy in this patient group [41]. False-positive results can occur as the result of cross-reactions with other systemic mycoses [42] and are documented in TB patients [43]. In The Gambia, crop contamination with *Aspergillus* spp. has been documented, resulting in chronic aflatoxin exposure [44]. Cross-reactions with *Aspergillus* might result in false-positive results on LAT. The increased likelihood of false-negatives and false-positives in HIV-positive and active TB patients, respectively, reduces confidence in the seroprevalence estimate presented by this study.

### Future Research Directions

Future research should examine the burden of *Histoplasma* in larger sample populations than presented by this study to increase the accuracy and reliability of outcomes.

Further examination of the effect of coinfections, including HIV and TB, on anti-*Histoplasma* antibody detection by LAT is required. Determining patient immunocompetence at presentation will help to determine the suitability of the LAT as an immunodiagnostic and support the interpretation of seroprevalence estimates. Crucially, definitive diagnostic techniques, including *Histoplasma* antigen detection tests and PCR, should be utilized to provide greater test sensitivity in people with HIV [41, 45].

The following future research directions in The Gambia are proposed: (i) robust observational studies to determine prevalence and incidence rates of *Histoplasma* infection in defined sample populations and to explore the demographic, clinical, and environmental risk factors for exposure and infection; (ii) phylogenetic characterization of *Histoplasma* isolates from human, animal, and environmental origins to determine the relationship of *Histoplasma* spp. in The Gambia to defined phylogenetic species globally; and (iii) qualitative studies to understand the awareness and educational needs of health care professionals with regards to histoplasmosis.

The proposed research directions will help to inform health care professionals and policy-makers on the prioritization of accessible and cost-effective diagnostic and treatment options for histoplasmosis and other fungal diseases of concern in The Gambia.

## CONCLUSIONS

The first evidence of *Histoplasma* exposure in active TB patients in The Gambia is demonstrated by this study. Due to the significant limitations of the sample size and use of the LAT in this patient group, robust *Histoplasma-*focused research is warranted in this context to accurately determine the burden of *Histoplasma* and to explore demographic, clinical, and environmental risk factors for exposure and infection. These activities would align with the WHO's first FPPL priority areas for action [1].
